# Supplementary material for: The cardiac autonomic response to acute psychological stress in type 2 diabetes
Source: PLoS One. 2022 Mar 18;17(3):e0265234. doi: 10.1371/journal.pone.0265234 (PMC8933038; doi:10.1371/journal.pone.0265234)
Supplement: S1 Fig — (PDF) [file pone.0265234.s001.pdf]

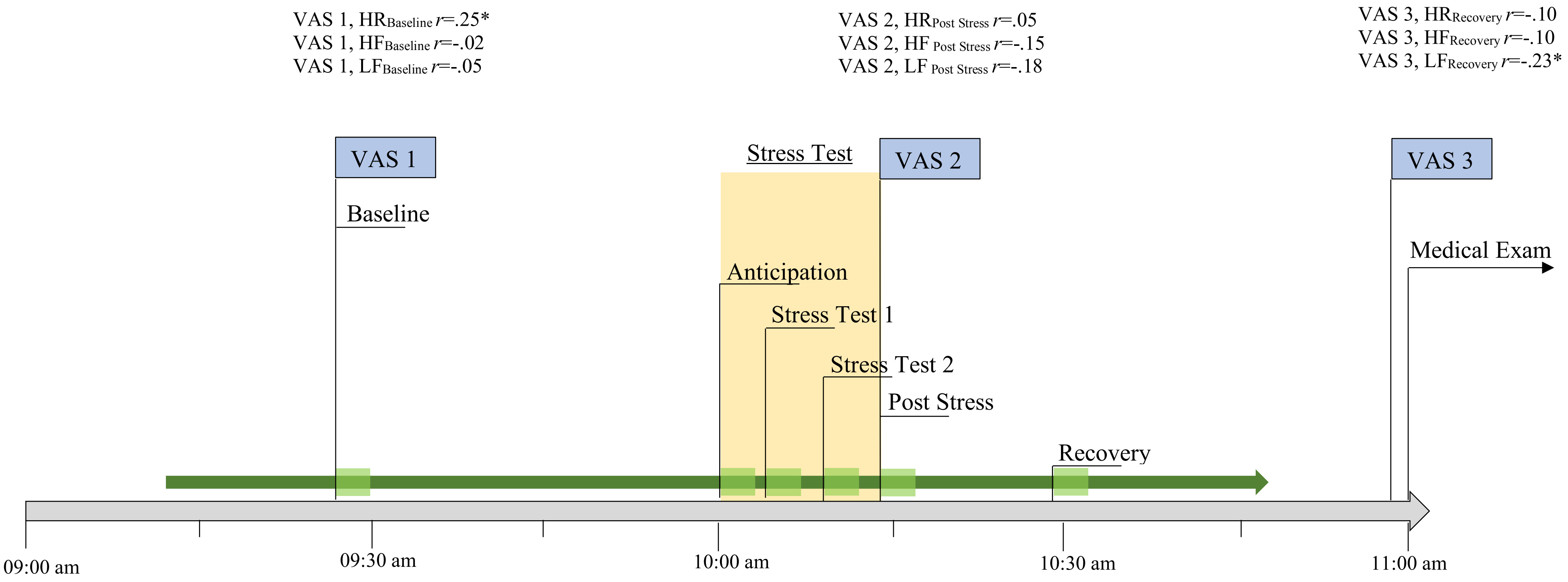

Fig. 6: Ideal-typical depiction of experimental procedure

ECG recording

3 min ECG sample

VAS 1 Visual analogue scale assessing psychological tension at baseline

VAS 2 Visual analogue scale assessing psychological tension directly after the stress test

VAS 3 Visual analogue scale assessing psychological tension 45 min after the stress test

Note: *p<.05; HR, HF and LF were log-transformed
