# Supplementary material for: The cardiac autonomic response to acute psychological stress in type 2 diabetes
Source: PLoS One. 2022 Mar 18;17(3):e0265234. doi: 10.1371/journal.pone.0265234 (PMC8933038; doi:10.1371/journal.pone.0265234)
Supplement: Attachment — Submitted filename: Response to Reviewers.docx [file pone.0265234.s002.docx]

**Reviewer #1**: ABSTRACT

*1. Line 22: The abbreviation for HRV should come from "heart rate variability".*

Thank you for pointing out this mistake. We have corrected it. (s. line 22)

*2. Line 27-28: Although the SDNN activity of diabetic patients was lower than that of the healthy group, there was no statistical difference in the response of SDNN and RMSSD to the stress test between the two groups. Therefore, the description of "impaired autonomic flexibility pattern" does not seem appropriate.*

Thank you. We have replaced the expression in question (s. line 27-28)

INTRODUCTION
This part is well written.

MATERIALS & METHODS
*1. Line 120-134, page 6-7: It may be easier for the readership if the authors provided a diagram of experimental procedure, so that the reader can more clearly know the time points of the six experimental phases. And confirm the time taken by the participants to complete the entire experiment. Was it 56 minutes (Line 225-227)? Moreover, it can also integrate the point-in-time correlation of various parameters such as HR, HRV, VAS, and TSST.*

Thank you for this suggestion. We have provided a supplementary figure depicting the experimental procedure including point-in-time correlations between the VAS and the physiological variables. As depicted in the figure, the study procedures took approximately 2 hours.

*2. Line 145-146, page7: Generally, the basic unit of HRV analysis, whether it is time domain analysis or frequency domain analysis, is based on a 5-minute period. Why is the 3-minute ECG record used here for analysis? Please explain the reason.*

We used 3-min intervals to achieve a more detailed assessment of the stress response during the stress test. The stress test lasted only approximately 14 minutes and we aimed for 3 samples assessing the HR and HRV response to stress anticipation, the speech task and the surprise arithmetic task. Due to participants varying reactions to the stress test, there was a (small) variance in the exact timing of the stress test. Using 3-minute samples ensured three non-overlapping ECG samples for all participants. To ensure comparability, we wanted to keep all ECG-samples the same length and therefore only used 3-minute samples.

We have inserted a short explanation of the ECG sample length in the Methods section (s. lines 151-152).

As suggested in your third comment below, we have replaced RMSSD and SDNN with HF and LF HRV. For both HF and LF HRV, samples of <5 min. have been shown to be reliable.

See for example:

Castaldo, R., Montesinos, L., Melillo, P., James, C., & Pecchia, L. (2019). Ultra-short term HRV features as surrogates of short term HRV: a case study on mental stress detection in real life. *BMC medical informatics and decision making*, *19*(1), 1-13.

Baek, H. J., Cho, C. H., Cho, J., & Woo, J. M. (2015). Reliability of ultra-short-term analysis as a surrogate of standard 5-min analysis of heart rate variability. *Telemedicine and e-Health*, *21*(5), 404-414.

Salahuddin, L., Cho, J., Jeong, M. G., & Kim, D. (2007, August). Ultra short term analysis of heart rate variability for monitoring mental stress in mobile settings. In *2007 29th annual international conference of the ieee engineering in medicine and biology society* (pp. 4656-4659). IEEE.

*3. Line 156-160, page8: Generally, time-domain analysis is more commonly used to analyze periodic or longer-period records. Frequency domain analysis seems to be more commonly used to observe autonomic changes in a short period of time. It also uses 3-5 minutes as the unit for analysis, which seems to be more in line with the author's design of 6 sampling and 3-minute analysis (Line 1458-146). In addition, frequency domain analysis can additionally observe changes in sympathetic activity, because according to the literature, the sympathetic activity of diabetic patients will also be affected. Based on the above two factors, it is suggested that the author can try to observe the results of this research data by frequency domain analysis.*

We have replaced RMSSD and SDNN with HF and LF power. RMSSD was highly correlated with HF power and SDNN with LF power and our results did not change when using frequency domain analysis (s. results section). Correlations and results of both frequency and time-domain analysis can be compared below on page 5 and 6 of this document.

RESULTS
Overall, this section reads well.

DISCUSSION
*1. Line 260-261, page 15: The data in Figures 3 and 4 cannot support the description of “impaired autonomic flexibility”. Because there was no difference in HRV response between the two groups after the TSST test.*

Thank you. We have replaced the expression in question (s. line 281).

*2. Line 263, page 15: What does "Cardiac response pattern" refer to? The meaning here is unclear, please specify exactly which physiological parameter response?*

Thank you for pointing this out. We have revised the sentence. (s. line 281)

*3. Line 277-279, page 16: This sentence means that age and diabetes have a bottoming effect on the reduction of RMSSD activity. Is there any evidence in the literature? Isn't their influence cumulative?*

As we found no conclusive evidence for neither a floor effect, nor a cumulative effect, we revised the section deleted the sentence.

*4. Line 310-313, page 17: The effects of exercise training and breathing exercise intervention on autonomic flexibility mentioned in this paragraph seem to be irrelevant to this study?*

Thank you. We have deleted the paragraph in question.

CONCLUSION
*The conclusions of the paper were very general and deserve better specificity. The conclusion of the paper should say more about how to use this information to make a change in practical application, and comment on the specific research that is suggested to move the science forward.*

Thank you for your suggestion. We expanded the respective paragraphs (s. lines 334-339 and 345-347).

***Reviewer #2****:*

*1. In the subsection “Participants”, the number of the participants and their demographic data should be clearly described. Also, the exclusion criteria were not well described.*

Thank you for pointing this out. We have revised the section (s. lines 78-81).

Number of participants and demographic data can be found in the Results section (sample description, s. lines 211-215 and table 1). We have expanded this section as well.

*2. For the subsection “ECG Sampling and HRV Analysis”, the major concerns are as follows.
(1) The authors used SDNN and RMSSD of HRV to indicate the “sympathovagal balance” and “cardiac vagal outflow”, respectively. How about the frequency domain indices of HRV?*

We have replaced RMSSD and SDNN with HF and LF power. RMSSD was highly correlated with HF power and SDNN with LF power and our results did not change when using frequency domain analysis (s. Results section). Correlation and results of both frequency and time-domain analysis can be compared on page 5 and 6 of this document.

*(2) What types of signals were recorded using the ECG logger? The ECG signals or “Heart rate and heart rate variability samples”?*

Thank you for pointing out this inaccuracy. We have revised the paragraph in question (s. line 144-145).

*(3) Why a sampling frequency of 8000 Hz was used?*

The ECG system we used uses oversampling to improve resolution and signal-to-noise ratio. We have included an explanation in the respective section (s. line 145-146).

*(4) The positions of five ECG electrodes.*

Thank you for your suggestion. We have included a description of the placement of the electrodes in line 146-148.

*(5) How to perform the procedure “artifact correction”?* what does the term “artifact rate” mean? (line 155)

Thank you for your suggestion. We have expanded this section and included a more detailed description of the procedure (s. lines 160-170).

*3. The “Results” section was not well organized.*

Thank you for pointing this out. We have reorganized the results section. Results on HR and HRV are now placed before the results on psychological tension.

*4. There are a lot of incorrect or inappropriate expressions. Only in the abstract, the meanings of many terms such as “Multilevel analyses”, “attenuated HR”, “decreased HR curvature”, “HR recovery”, and “stress system” are ambiguous.*

Thank you for pointing this out. We have tried to be more specific (s. lines 26-29, 34-35, 189, 230, 279, 281)

| **Results for Time- vs. Frequency Domain Analysis**  **Table 3**: Multilevel Model on **Log(HF HRV):** Estimates of Fixed Effects | | | | |
| --- | --- | --- | --- | --- |
| Parameter | *Estimate* | *SE* | *t* | *p* |
| Intercept | 1.69 | 0.17 | 10.13 | <.001 |
| Time (linear) | -0.14 | 0.09 | -1.62 | .11 |
| Time (quadratic) | 0.04 | 0.02 | 2.25 | .03 |
| Type 2 Diabetes | -0.03 | 0.12 | -1.71 | .09 |
| Type 2 Diabetes* Time (linear) | 0.03 | 0.06 | 0.47 | .64 |
| Type 2 Diabetes* Time (quadratic) | -0.004 | 0.01 | -0.30 | .77 |
| Note: Effects of age, gender, BMI and hypertensive Medication were controlled for. | | | | |

| **Table 3**: Multilevel Model on **Log(RMSSD**): Estimates of Fixed Effects | | | | |
| --- | --- | --- | --- | --- |
| Parameter | *Estimate* | *SE* | *t* | *p* |
| Intercept | 1.13 | 0.09 | 13.05 | <.001 |
| Time (linear) | -0.09 | 0.45 | -2.05 | .04 |
| Time (quadratic) | 0.02 | 0.01 | 2.85 | .01 |
| Type 2 Diabetes | -0.09 | 0.06 | -1.40 | .16 |
| Type 2 Diabetes* Time (linear) | 0.01 | 0.03 | 0.26 | .79 |
| Type 2 Diabetes* Time (quadratic) | -0.001 | 0.01 | -0.14 | .89 |
| Note: Effects of age, gender, BMI and hypertensive Medication were controlled for. | | | | |

Correlation Between Log(HF HRV) and Log(RMSSD):

Baseline: *r*=.96, *p*<.001

Anticipation: *r*=.95, *p*<.001

Stress Test 1: *r*=.94, *p*<.001

Stress Test 2: *r*=.94, *p*<.001

Post-Stress: *r*=.96, *p*<.001

Recovery: *r*=.96, *p*<.001

| **Table 4**: Multilevel Model on **Log(LF HRV):** Estimates of Fixed Effects | | | | |
| --- | --- | --- | --- | --- |
| Parameter | Estimate | SE | t | p |
| Intercept | 2.34 | 0.17 | 14.23 | <.001 |
| Time (linear) | -0.04 | 0.09 | -0.43 | .67 |
| Time (quadratic) | 0.02 | 0.02 | 1.12 | .24 |
| Type 2 Diabetes | -0.27 | 0.06 | -2.20 | .03 |
| Type 2 Diabetes* Time (linear) | 0.11 | 0.06 | 1.79 | .08 |
| Type 2 Diabetes* Time (quadratic) | -0.02 | 0.01 | -1.86 | .06 |
| Note: Effects of age, gender, BMI and hypertensive Medication were controlled for. | | | | |

| **Table 4**: Multilevel Model on **Log(SDNN)**: Estimates of Fixed Effects | | | | |
| --- | --- | --- | --- | --- |
| Parameter | Estimate | SE | t | p |
| Intercept | 1.27 | 0.08 | 16.52 | <.001 |
| Time (linear) | -0.03 | 0.04 | -0.72 | .47 |
| Time (quadratic) | 0.01 | 0.01 | 1.59 | .11 |
| Type 2 Diabetes | -0.12 | 0.06 | -2.17 | .03 |
| Type 2 Diabetes* Time (linear) | 0.05 | 0.03 | 1.66 | .10 |
| Type 2 Diabetes* Time (quadratic) | -0.01 | 0.01 | -1.57 | .12 |
| Note: Effects of age, gender, BMI and hypertensive Medication were controlled for. | | | | |

Correlation Between Log(LF HRV) and Log(SDNN):

Baseline: *r*=.97, *p*<.001

Anticipation: *r*=.97, *p*<.001

Stress Test 1: *r*=.98, *p*<.001

Stress Test 2: *r*=.98, *p*<.001

Post-Stress: *r*=.98, *p*<.001

Recovery: *r*=.97, *p*<.001
