# Supplementary material for: The cardiac autonomic response to acute psychological stress in type 2 diabetes
Source: PLoS One. 2022 Mar 18;17(3):e0265234. doi: 10.1371/journal.pone.0265234 (PMC8933038; doi:10.1371/journal.pone.0265234)
Supplement: Attachment — Submitted filename: Response to Reviewers.docx [file pone.0265234.s003.docx]

**Rebuttal Letter**

*LINE 28 & 279: The sentence described in the text (Line 28 & 279) appears to be misleading: "This pattern was accompanied by significantly decreased low frequency HRV ..... between the groups."
The term "decreased" to describe the LF HRV response in the text appears to be misleading. Because there was no difference in LF HRV in group-by-time interaction in the stress response, only the LF HRV in the DM group was consistently lower than that in the healthy group. Therefore, it is suggested that the word "lower" is more appropriate to replace "decrease" here.*

Thank you for your suggestion! We have replaced the word “decreased” with the word “lower” in Line 28 and 279. *LINE 32-33 & 341-342: "The impairment of the autonomic nervous system commonly found in people with type 2 diabetes likely affects the stress response." This sentence in the conclusion is too excessive and vague. In fact, the difference in ANS was only in the LF, and the stress response only affected the heart rate and subjective perception, however, there was no significant difference in the ANS response. Therefore, it is recommended to make more specific and precise conclusions to avoid misunderstandings.*

Thank you! We have replaced the sentence in question with a more precise conclusion. See lines 32-34 & 342-344
